# Neurocognitive Mechanisms Underlying Internet/Smartphone Addiction: A Preliminary fMRI Study

**DOI:** 10.3390/tomography8040150

**Published:** 2022-07-11

**Authors:** Suk Won Han, Cheol Hwan Kim

**Affiliations:** Department of Psychology, Chungnam National University, Daejeon 34134, Korea; cheol.hwan.kim2391@gmail.com

**Keywords:** attention, smartphone addiction, fMRI

## Abstract

The present study investigated the neurocognitive mechanisms underlying smartphone/internet addiction. We tested a specific hypothesis that the excessive, uncontrolled use of smartphones should be related to the ability of controlling attention in a purely endogenous and self-regulatory manner. In an fMRI experiment, in which 43 adults participated, we had participants detect and identify specified target stimuli among non-targets. In some trials, 10 s oddball movies were presented as distractors. While the participants try to filter out the distractors and focus their attention on the main task, the activation profiles of the frontoparietal brain regions were examined. The results showed that the people with a higher risk of being addicted to smartphone use failed to filter out distractors via the endogenous control of attention. The neuroimaging data showed that the high-risk group showed significantly lower levels of activation in the frontopolar cortex (FPC). We conclude that people at a high risk of smartphone addiction have difficulty endogenously shifting their attention from distracting stimuli toward goal-directed behavior, and FPC plays a critical role in this self-regulatory control of attention.

## 1. Introduction

Internet/smartphone addiction refers to addicts’ excessive and uncontrolled use of smartphones. Recently, this issue has received growing attention, being considered as a serious public health issue [1,2,3]. In response, a growing number of studies have been carried out to clarify the psychological and behavioral mechanisms underlying smartphone addiction. While it is widely presumed that impaired cognitive control is associated with the addicts’ inability to suppress behavioral impulses to use smartphones [4], the specific nature of the functional relationship between smartphone addiction and cognition remains unclear [3,5].

A core function of the human cognitive system is to configure a queue of discrete processes and behaviors to enable one to flexibly adapt to a dynamically changing environment [6]. Specifically, in the face of a cognitively demanding task, a task set is formulated such that people focus their attention on the task-related stimuli, filtering out other task-irrelevant sensory events. By this, a limited amount of processing resources is optimally allocated among multiple stimuli. However, the abrupt appearance of a novel, salient, or intriguing stimulus powerfully captures and takes attention away from the ongoing task. If the stimulus that captured attention is evaluated to be distracting, attention should be deviated from the stimulus and redirected to the original task.

It is hard to override attentional capture by a salient distractor. What matters is how effectively one reorients attention from the attention-capturing distractor to the ongoing task. This endogenous, self-regulatory control of attention is crucial in daily lives. Recently, we developed an experimental paradigm to isolate the neural substates underlying the purely endogenous and self-regulatory control of attention [6]. Notably, in many previous studies investigating the endogenous control of attention, the endogenous shifting of attention was initiated by an abrupt onset of attention-capturing stimuli [7,8,9]. Contrary to this, the recently developed paradigm requires participants to shift their attention from attention-capturing, distracting stimuli to the main task without any sensory transitions, such as an abrupt onset or offset of a stimulus. Specifically, in each 24 s long trial, the participants were required to detect and identify multiple target stimuli—letters embedded in a rapid serial visual presentation of digits (RSVP). In a subset of trials, a 16 s long movie stimulus was simultaneously presented with the RSVP. The abrupt onset of the movie would capture attention. However, during the movie presentation lasting 16 s, the participants would have to shift their attention to the task-related stimuli for the successful completion of the task. Importantly, at the moment the participants shifted their attention, the movie was still being presented with no abrupt sensory change. Hence, the shifting of attention is not accompanied by any salient sensory event but triggered by one’s own endogenous control of attention.

In the present fMRI study, using this paradigm to probe the purely endogenous, self-regulatory control of attention, we explored the neurocognitive mechanisms associated with internet/smartphone addiction. To do so, a smartphone addiction measure scale, a self-report questionnaire, was administered via a computer to rate the extent to which the participants are at risk of being addicted to smartphone usage [10]. Then, the participants were asked to perform a cognitive task involving the process of focusing one’s attention on a task-relevant stimulus while inhibiting a distractor in an fMRI scanner.

To analyze the data, we examined the behavioral task performance to investigate how the degree of smartphone addiction risk is related to the cognitive process of suppressing ongoing distractions and focusing on the main task. At the same time, we examined which brain regions are associated with the degree of smartphone addiction risk. As regions of interest (ROI), we selected the core brain regions included in the multiple demand network and default mode network [11,12,13,14,15,16]. Specifically, the anterior insula (AI), anterior cingulate (ACC), lateral prefrontal cortex (LPFC), frontal eye fields (FEF), and intraparietal cortex (IPS) in the multiple demand network and the frontopolar cortex (FPC) and temporo-parietal junction (TPJ) in the default mode network were probed. These regions were chosen among many other regions because they were found to form not only functional networks but also a structural cortical network, mediating executive control [17]. We examined how these regions respond to the presentation of oddball movie stimuli and whether their responses differ depending on each individual’s risk of being addicted to smartphone usage.

## 2. Materials and Methods

### 2.1. Participants

A total of 43 adults (26 females, aged 23–33) participated in the study for monetary compensation. Twenty participants’ data were also reported in our previously published paper [6]. The Chungnam National University Institutional Review Board approved the experimental protocol, and written informed consent was obtained from each participant. The study was performed in accordance with the approved guidelines.

### 2.2. Behavioral Paradigm

**Figure 1:**
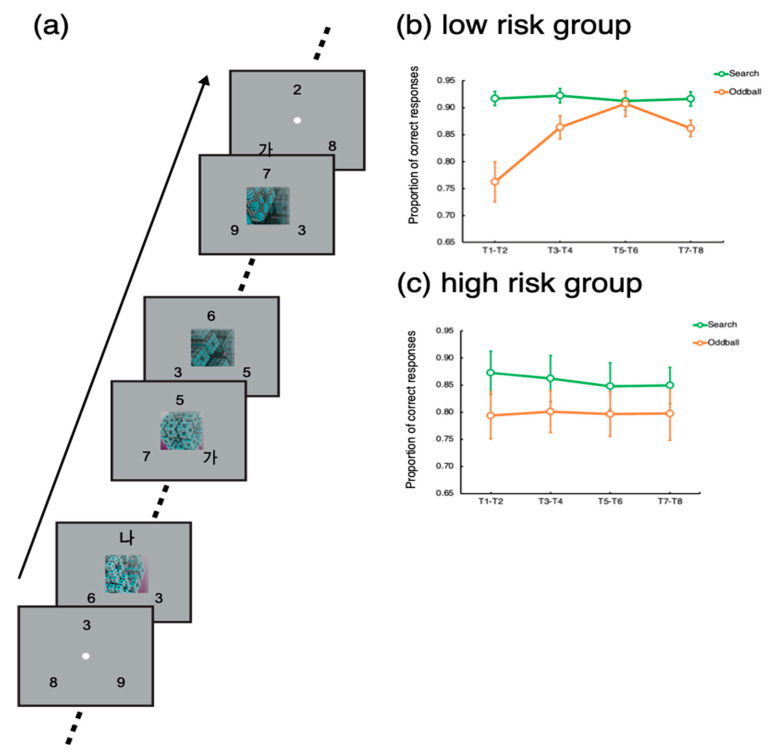
Trial design and behavioral results. (**a**) Trial design. (**b**) Behavioral results of the low-risk group participants. (**c**) Behavioral results of the high-risk group participants. Error bars represent standard errors of the mean.

The experiment was designed and run using Psychopy [18]. The experimental protocol is also described elsewhere [6]. As shown in Figure 1a, the task was to detect and identify Korean letters embedded in three rapid serial visual presentations (RSVP) of digits. Each RSVP was presented on three locations evenly spaced on an imaginary circle, whose radius was 5 degrees of a visual angle. Each frame in the RSVPs lasted 200 ms—that is, the individual letters and numbers were shown for 200 ms. The target letters (‘가’, ‘나, ‘다’, or ‘라’) could be presented in any of the three RSVPs. When a target was presented, the participants indicated which of the four letters appeared by pressing one of the four designated buttons in the scanner. A single trial, which included eight targets, lasted 24 s. Each target stimulus was separated by a 2 or 4 s interval, during which distractors were presented. The inter-target interval was randomized such that it was difficult to predict exactly when a target would appear.

Importantly, in 24 trials out of a total of 96 trials, an oddball movie lasting 16 s was presented at the center of the screen 4 s after the trial onset, simultaneously with the RSVPs (Oddball trials). The set of oddball stimuli comprised movies depicting non-meaningful, abstract animations (e.g., continuously transforming fractals, molecular polymerization, swirling waves, constantly rotating color patches in random directions, evolving line drawings of geometric shapes, dynamically transforming objects, moving flashlight in random direction, or continuously evolving colored geometric shapes) and movies depicting real-world situations (fast-moving roller coasters, overturning ships, moving toys, a remote-controlled vacuum cleaner, a dogfight of jet-fighters, online gaming, and a laptop commercial). The abstract oddballs and real-world oddballs did not yield differential results [19]. The remaining 72 trials did not include any oddball (Search trials). The order of the different trial types was randomized such that participants could not predict which trial type would be presented.

In each trial lasting 24 s, a total of eight targets (T1~T8) were presented with 2–4 s of inter-target intervals. To assess the participants’ task performance for the search and oddball trials, we calculated the proportions of correct responses for each target (T1~T8). To increase statistical power, the target accuracies for each consecutive pair of targets (T1 − T2, T3 − T4, T5 − T6, and T7 − T8) were averaged. Then, we applied *t*-tests to compare the target accuracy between the search and oddball trials. Notably, T1 could appear 2 s or 4 s after the trial onset for the search trials, while for the oddball trials, T1 was presented 4 s after the trial onset, simultaneously with the oddball onset.

Prior to the fMRI experiment, the participants completed a questionnaire designed to measure an individual’s risk of being addicted to smartphone usage. The participants rated 18 items regarding their smartphone usage habits, their psychological experiences when abstinent from smartphone usage, and the impacts of smartphone usage on their daily lives. They responded based upon a Likert-type scale, ranging from 0 (never or rarely) to 3 (very often).

### 2.3. fMRI Methods

All the imaging parameters and preprocessing steps were identical to those of our previous work [19]. Specifically, anatomical 2D and 3D high-resolution T1-weighted images were acquired with conventional parameters on a 3T Philips scanner at the Korea Basic Science Institute. For the functional scan, thirty-three 3.5 mm axial slices (0.5 mm skip; 3.75 × 3.75 mm in-plane) were taken parallel to the AC-PC line (TR, 2000 ms; TE, 35 ms; FA, 79^°^; FOV, 240 mm) for a total of 255 brain volumes per fMRI run. There were five or six functional runs, each of which included sixteen trials. A blank interval of variable duration following an exponential distribution (9 trials × 4 s, 5 trials × 8 s, 2 trials × 12 s) was inserted between each trial.

The imaging data were analyzed using FSL (http://fsl.fmrib.ox.ac.uk). The data preprocessing included non-brain removal to improve image registration, slice scan time correction, 3D motion correction, high-pass filtering (100 s period cutoff), and spatial smoothing with a 5 mm Gaussian kernel (FWHM). All these parameters were set by default in FSL and commonly applied to previous event-related fMRI studies [20,21,22]. Following these preprocessing steps, all the functional data of each participant were co-registered to each individual’s anatomical T1-weighted image and transformed into the Montreal Neurological Institute standard brain.

**Figure 2:**
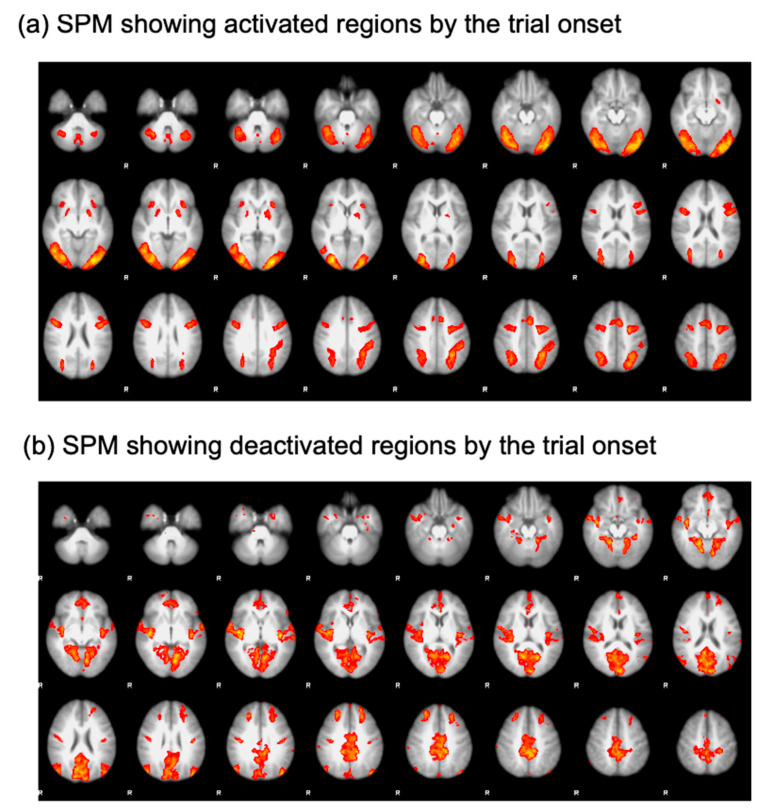
SPM results. (**a**) Regions activated by the trial onset. (**b**) Regions deactivated by the trial onset.

To isolate the ROIs, a generalized linear model analysis was performed. Specifically, regressors were defined for each trial type (search and oddball trials) and convolved with a double-gamma function implemented in FSL [23]. Then, an open contrast was run by assigning a coefficient of 1 to all the regressors. This contrast was employed to find regions activated by the onset of the trials (Figure 2a). The resulting statistical parametric map (SPM) was thresholded using clusters determined by a voxelwise Z threshold of 2.3 and a cluster significance threshold of *p* = 0.05 corrected for whole-brain multiple comparisons. This SPM analysis yielded significant activational foci on the core nodes of the multiple demand network comprising the lateral prefrontal cortex (LPFC), anterior insula (AI), anterior cingulate cortex (ACC), frontal eye fields (FEF), and intraparietal sulcus (IPS). To define ROIs in the default mode network, a coefficient of −1 was assigned to all the regressors to find regions deactivated as the trial started (Figure 2b). Using this contrast, we were able to define the frontopolar cortex (FPC) and temporal-parietal junction (TPJ) [11,24,25,26]. ROIs were drawn as a sphere with a radius of 8 mm and centered on the peak coordinates of the activational foci. Details of the ROIs are presented in Table 1.

For the ROI analyses, the event-related time course of the blood-oxygenated-level-dependent (BOLD) signal for each participant and trial type was calculated and plotted as the percent signal change relative to the mean of the run. The activation time courses were averaged across participants, yielding group-averaged time courses. As no hemispheric difference was found, the time courses of the bilateral ROIs were collapsed to increase power [19,27].

To statistically assess the difference in activity evoked by the oddball distractor presentation, we averaged the signal amplitudes of eight volumes (16 s), starting from 4 s after the oddball onset for each ROI. These averaged signal amplitudes over eight volumes were compared across the groups via two-sample t-tests. The resulting p-values were divided by the number of ROIs (7, LPFC, AI, ACC, IPS, FEF, FPC, and TPJ) for multiple comparisons correction (Bonferroni correction)

## 3. Results

### 3.1. Behavioral Results

**Figure 3:**
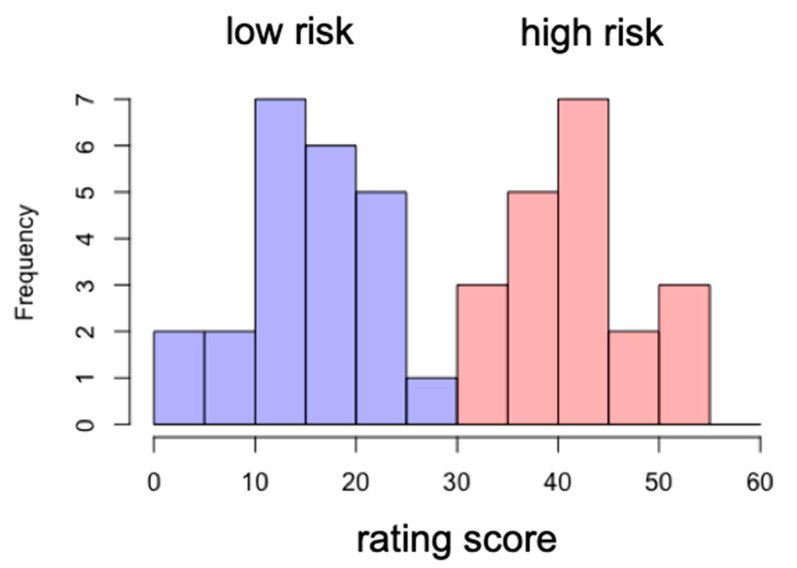
Distribution of rating scores for low-risk and high-risk groups.

Based upon the smartphone addiction rating scale score, we divided the participants into two groups: low-risk and high-risk groups. The cutoff score was 33. The participants whose score was below 33 were assigned to the low-risk group, while the ones who scored above 33 were assigned to the high-risk group. The mean and standard deviation of the high-risk group score were 42 and 5.9, respectively, while those for the low-risk group score were 15 and 6.5 (see Figure 3).

For the low-risk group, target accuracy was significantly lower for the oddball trials for the target pair of T1–T2, t(22) = 4.41, *p* < 0.001. Significant distractor interference persisted until the target pair of T3–T4, t(22) = 2.85, *p* < 0.01. Then, for the target pair of T5–T6, there was no difference in target accuracy between the oddball and search trials, *p* > 0.67. For the last pair, there was a significant difference, but its magnitude was smaller than that for the initial target pairs, *p* < 0.005.

For the high-risk group, the target accuracy for the search trial was significantly worse than that for the oddball trials for the target pairs of T1–T2, T3–T4, and T5–T6, t’s > 2.37, p’s < 0.05. For the last target pair (T7–T8), the difference was not significant, *p* > 0.19, but the task performance for the last target pair was not greater than the performance for the initial targets, *p* > 0.92.

These results suggest that the low-risk participants’ attention was captured by the onset of the oddball movie presentation interfering with the task performance. However, the worsened performance recovered while the distraction was going on. Notably, the performance for the last target pair (T7–T8) worsened. As a reviewer suggested, this might be because the offset of the movie caused perceptual interference for this group of participants. However, for the high-risk participants, such a recovery was not observed; their attention was captured and captivated by the movie distractors. These results suggest that a high risk of addiction to smartphone usage is related to the endogenous control of attention filtering out distractors and keeping focus on the main task.

### 3.2. Imaging Results

**Figure 4:**
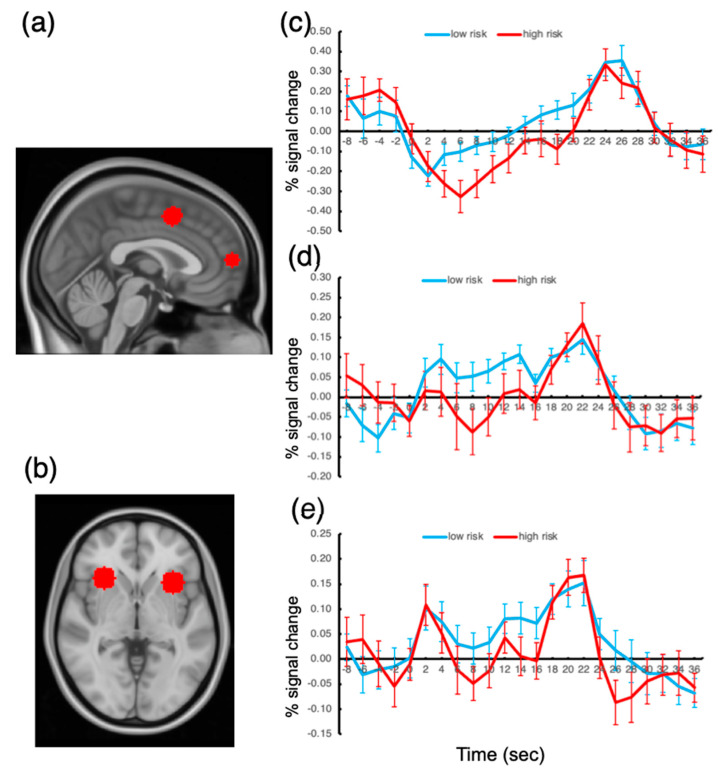
Anatomical locations of the ROIs and activation time courses. (**a**) FPC and ACC ROIs. (**b**) AI ROI. (**c**) Activation time course for FPC. (**d**) Activation time course for ACC. (**e**) Activation time course for AI. Error bars represent the standard errors of the mean.

The responses of the ROIs to the presentation of the oddball stimuli were examined, and the activation amplitudes were compared across the low- and high-risk groups. To assess the group difference in oddball responses, we extracted activation time courses during the 10 s oddball presentations and determined the activation amplitude by averaging the fMRI signal from the oddball onset volume (time 0 at the time course plot; see Figure 4) to the volume at time 20. These averaged signal amplitudes were compared between the groups for each ROI.

The results showed that, among all the ROIs, the FPC response to the oddball presentation was significantly greater for the low-risk group than that for the high-risk group, t(41) = 3.04, *p* < 0.05, Bonferroni-corrected. The ACC and AI responses also showed greater activation amplitudes for the low-risk group, but the difference failed to reach significance, p’s > 0.10. We also probed the other ROIs but found no significant difference between the groups.

These results suggest that the FPC activation amplitude under sustained distractor interference is related to the degree of risk of being addicted to smartphone use. We found no activation difference in the other ROIs. This finding explains why there has been so much controversy regarding the brain substrates of smartphone addiction; the extensive regions of the frontoparietal cortex did not show significantly different activation depending on the degree of risk for being addicted to smartphone usage.

## 4. Discussion

The present study investigated the behavioral and neurocognitive characteristics of smartphone addiction. We found that people with a high risk of smartphone addiction had impaired cognitive control. Specifically, these people were found to have difficulty in shifting their attention from attention-capturing, distracting stimuli to an impending task to be performed. Such attentional shifting should take place in a purely endogenous, self-regulatory manner.

The contribution of the present study is to identify a specific cognitive function associated with smartphone addiction. While a large body of studies has been devoted to understanding which cognitive processes and abilities are related to smartphone addiction, this issue is yet to be clarified. A reason for this is that many previous studies did not set a specific, a priori hypothesis but explored several possibilities without a strong background. Specifically, researchers simply posited that people with smartphone addictions should have difficulty in terms of cognitive control. To test this hypothesis, paradigms such as the Stroop task, Eriksen Flanker task, or Go/No-Go task were employed. While these are excellent and reliable paradigms to study cognitive control, it is not well established that the cognitive processes underlying these tasks are related to smartphone addiction. In recent studies, researchers found no significant difference in the cognitive process for these tasks between the smartphone addiction group and the control group [4,20,28,29]. With the lack of behavioral distinction, any difference in the event-related potential data or fMRI functional connectivity is difficult to interpret.

Contrary to previous studies, we targeted a specific cognitive function: the endogenous, self-regulatory control of attention. We surmised that people with a high risk of smartphone addiction would show a similar cognitive ability with people with a low risk in the domains of the top-down and bottom-up orienting of attention and task performance initiated by transient sensory events. This proposition fits well with the extant studies, showing that the control group and addiction group showed equivalent performances in a variety of cognitive tasks [4,28,29,30]. Instead, we hypothesized that it is the process of shifting/reallocating attention from salient distractors toward goal-directed task stimuli that is associated with smartphone addiction; a common feature of addicted people is that they cannot deviate their attention away from smartphones.

To test this hypothesis, we employed a recently developed experimental paradigm, straining one’s endogenous, self-regulatory control of attention [6]. Our behavioral data clearly showed that the high-risk individuals for smartphone addiction had difficulty shifting their attention away from distracting stimuli that captured attention; their attention was captivated by the distractor while it persisted. However, the low-risk individuals were able to shift their attention away from the distractor and refocus on the main task by their own will.

With this significant behavioral difference, we found that the frontopolar cortex activity of the high-risk people was significantly weaker than that of the low-risk people. The role of the frontopolar cortex in the present experimental setting is remarkable given that an influential theory emphasized the role of this region in managing competing goals, in part by keeping track of the importance of current and alternative goals, and therefore in enabling switching away from ongoing behavior [31].

Furthermore, while the present FPC is included in the default mode network, recent evidence shows that this region has a wider role than previously presumed such that a variety of executive and social cognitive processes have been associated with this region [16,26]. Specifically, it was argued that the FPC plays a crucial role in maintaining a long-term, behavioral goal, while other frontoparietal regions are involved in the stage-specific cognitive process of a task [16].

Given that the endogenous, self-regulatory control of attention should be placed at the top of the functional hierarchy of the human brain and that it is this process that is responsible for mitigating the problems caused by smartphone addiction, it makes sense that the FPC activation level was significantly different depending on the risk of smartphone addiction.

Finally, it is noteworthy that the AI and ACC showed a similar trend with the FPC, though the activation difference observed in these regions did not reach a significant level. A recent study showed that people prone to alcohol addiction showed poor inhibitory control, which was associated with less activity of the insular and supplementary motor area, which were close to the present AI and ACC [32]. The present results fit well with this study because the current results imply that the low-risk people developed the inhibitory control over time better than the high-risk people.

To conclude, we found that a crucial cognitive process associated with smartphone addiction is the endogenous, self-regulatory control of attention, which is initiated by one’s own will without any aid from transient sensory events such as the abrupt onsets of stimuli. At the same time, the activation level of the frontopolar cortex was found to be related to the degree of risk for being addicted to smartphone usage.

## Figures and Tables

**Table 1:** Coordinates and Z-values of ROIs. DMN—Default Mode Network, MDN—Multiple Demand Network.

ROI Name	Z-Value	MNI152 Coordinates		
		x	y	z
DMN ROI				
FPC	5.86	0	58	10
Right TPJ	3.55	58	−42	20
Left TPJ	3.52	−60	−46	20
MDN ROIs				
Right ACC	4.93	8	12	50
Left ACC	5.99	−6	8	48
Right AI	4.94	30	22	−2
Left AI	4.95	−32	18	−2
Right FEF	3.89	26	0	52
Left FEF	6.02	−26	−4	46
Right IPS	5.02	32	−54	42
Left IPS	6.00	−24	−68	42
Right LPFC	5.99	46	12	24
Left LPFC	6.07	−42	2	24

## Data Availability

All the data will be publicly shared upon the acceptance of the article.
